# Ultrahigh-Temperature Regeneration of Long Period Gratings (LPGs) in Boron-Codoped Germanosilicate Optical Fibre

**DOI:** 10.3390/s150820659

**Published:** 2015-08-20

**Authors:** Wen Liu, Kevin Cook, John Canning

**Affiliations:** 1 *i*nterdisciplinary Photonics Laboratories (*i*PL), School of Chemistry, The University of Sydney, Sydney NSW 2006, Australia; E-Mails: liusenanbei@gmail.com (W.L.); kevin.cook@sydney.edu.au (K.C.); 2 College of Optoelectronic Science and Technology, National University of Defense Technology, Changsha 410073, China

**Keywords:** regenerated gratings, long period gratings, fibre optic sensors, strain sensitivity, temperature dependence, transition temperature, melting temperature, glass relaxation

## Abstract

The regeneration of UV-written long period gratings (LPG) in boron-codoped germanosilicate “W” fibre is demonstrated and studied. They survive temperatures over 1000 °C. Compared with regenerated FBGs fabricated in the same type of fibre, the evolution curves of LPGs during regeneration and post-annealing reveal even more detail of glass relaxation. Piece-wise temperature dependence is observed, indicating the onset of a phase transition of glass in the core and inner cladding at ~500 °C and ~250 °C, and the melting of inner cladding between 860 °C and 900 °C. An asymmetric spectral response with increasing and decreasing annealing temperature points to the complex process dependent material system response. Resonant wavelength tuning by adjusting the dwell temperature at which regeneration is undertaken is demonstrated, showing a shorter resonant wavelength and shorter time for stabilisation with higher dwell temperatures. All the regenerated LPGs are nearly strain-insensitive and cannot be tuned by applying loads during annealing as done for regenerated FBGs.

## 1. Introduction

There is increasing demand for fibre gratings, both short and long period, to work in harsh and extreme environments where temperatures can exceed 1000 °C. Different methods have been employed to elevate the operable temperature of fibre gratings, including optimising glass composition [1], thermal stabilisation [2], forming type-II and type-IIA fibre gratings [3,4], hypersensitising through pre-irradiation [5], and, in particular, regenerating gratings [6,7]. Through regeneration, the normal type-I fibre Bragg grating (FBG) can withstand temperatures beyond 1200 °C [8], whilst the conventional type-I FBGs are optimised to operate below 80 °C for 20 years although they can be used for shorter periods at higher temperatures up to 300 °C [9]. Substantial research and commercialisation of regenerated fibre gratings has recently been carried out, including short term ultra-high temperature operation up to 1450 °C [10], while for non-regeneration involved FBGs, gratings need to be fabricated in fibre with special composition, such as sapphire, to get comparable operable temperature [11]. Very long lifetime operation of regenerated FBGs over 9000 h between 800 °C and 900 °C has also been reported [12]. Notably, the bulk of the work reporting and studying regeneration focuses on FBG regeneration, despite the regeneration process being a general glass process not confined to FBGs. Further, compared to FBGs structures such as long period gratings (LPGs) have greater sensitivity arising from coupling modes that are not spatially on top of each other. These make ideal filters with much wider rejection bands [13] which at room temperature have been used, for example, for gain equalization [14] and dispersion compensation [15]. Regenerated LPGs were only recently utilized as interferometry components for high temperature operation [16]; this is despite the fact that the characteristics of regenerated LPGs have not been studied. LPGs used for high temperature (~800 °C) applications can be fabricated by other means including laser writing (CO_2_ laser [17,18], CO laser [19] and femtosecond laser [20]) and electrical discharges [21,22]. By contrast, very few UV-written LPGs offer high thermal resistance, rarely exceeding 400 °C [19]. All these methods can benefit from regeneration and we explore that here focusing on UV-written structures.

In terms of applications, LPGs are used in many ways such as effective gain equalizers [14] and dispersion compensation [15], and also widely used in environment sensing, such as temperature, strain and refractive sensing. Regenerated LPGs will extend these applications into the ultra-high temperature regime ideally suited for harsh environments such as space, aircraft and vehicular engines and more. For example, regenerated LPGs are candidates for future chemical diagnostics through refractive index sensing in high temperature environments, such as within underground oil fields, which cannot be easily realized though ordinary optical sensing technologies, nor the non-regenerated LPGs or regenerated FBGs. Stable regenerated LPGs that do not move with temperature and strain are highly desirable because they offer a unique in-line ramp filter structure for intensity based spectral interrogators using swept narrow linewidth sources that outperform bulk devices and reduce the loses by being in-line. Such interrogators can perform robustly to probe regenerated fibre Bragg grating structures within the same robust environment such as that used to monitor diesel train engines [23]. By having weak structures, these regenerated LPGs can act as broadband attenuators and diverters distributed to provide equalized spatial information derived from time-of-light measurements along a fibre. 

In this paper, regeneration of UV-written LPGs within boron-doped germanosilicate fibre with a “W” refractive index profile is demonstrated. Both high fabrication reproducibility and high temperature stability in excess of 1000 °C are achieved. The behavior of the regenerated LPGs under different dwell temperatures at which regeneration occurs is compared, and the characteristic growth of the regenerated LPGs is investigated. Unlike FBGs which couple to identical backward and forward traveling modes, the LPG couples between the spatially distinct core fundamental modes and higher order cladding modes making it extremely sensitive to the processes which are thought to occur at the core/cladding interface. The special piece-wise linear temperature response this produces, compared with the regenerated FBGs fabricated in the same kind of fibre, coincides with measurable values of the transition temperature and melting temperature of glass in the fibre affected by the presence of stress, particularly at the various core/inner cladding/outer cladding interfaces. In addition to more specific industrial applications, the LPG provides the core probe for a new and novel instrumentation component that we will show is extraordinarily sensitive in investigating glass properties. LPG-based instrumentation is not new—we have previously reported a novel compact acoustic driven LPG viscometer with greater performance than many commercial instruments [24]. With these new regenerated LPGs it is now possible to undertake viscosity measurements in very hot industries including oil and smelting. These properties improves the basic understanding of composite glass systems overall as well as the principle of regeneration, areas of growing fundamental research and industrial importance.

## 2. Experiment Results 

Fibre grating regeneration is a glass milling process, involving index grating erasure and rebirth [8] through differences in local response to thermal annealing. For regenerated FBGs, type-I gratings form the “seed gratings”, describing both an index and phase periodic structure, and consequently a periodically varying local response along the grating with annealing. Although the seed grating is erased with temperatures over 600 °C, permanent physical relaxation is locked in the fibre, which will initiate the regeneration process. Similarly, a seed LPG, with index change below the damage threshold of glass, is another pattern of the glass history prerequisite for the realisation of regeneration.

### 2.1. Seed LPG Fabrication

**Figure 1:**
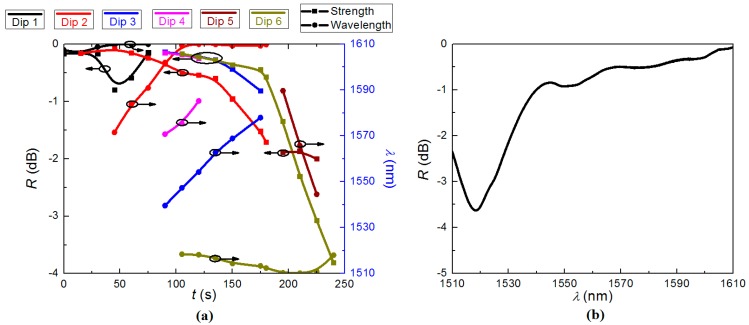
(**a**) The growth curve during the fabrication of the seed LPG; and (**b**) the transmission spectrum of the seed LPG.

Seed LPGs are fabricated in boron-codoped germanosilicate single mode fibre (core: [B_2_O_3_] ~ 20 mol%, [GeO_2_] ~ 33 mol%; inner cladding: [P_2_O_5_] ~ 11 mol%, [F] < 4 mol% ), which has a W-profile refractive index configuration, and can match the fibre *V* parameter of SMF 28 whilst retaining low loss and enhanced photosensitivity. The fibre used in this work is not commercially available but closely matches its successor the GF1 fibre (Photosensitive Single-Mode Low-NA Fiber) offered by Nufern (East Granby, CT, USA). The details of this fibre are listed at [25]. Before regeneration, the high temperature stability of the LPG in the single mode fibre without hydrogen loading was tested. The LPG was observed to erase completely above 600 °C and no regeneration was observed, proving the key role of hydrogen (or even an inert gas, e.g., helium [7]) loading, as it does in the FBG regeneration [6]. After loading hydrogen (H_2_: *P* = 180 bar, *T* = 80 °C, *t* = 2 days), four seed LPGs of length *L* = 35 mm were inscribed with a 193 nm ArF laser by direct writing through an amplitude mask (Λ = 390 μm). The repetitive rate of UV exposure is *RR* = 30 Hz, with pulse energy *E*_pulse_ ~ 10 mJ/cm^2^ and cumulative fluence *f*_cum_ ~ 72 J/cm^2^, respectively. The transmission spectrum of the LPGs was observed using an optical spectrum analyser (OSA: AQ6370B, Yokogawa, Sendagaya, Japan) with an erbium doped fibre amplifier (EDFA) over the spectral range (1510–1610) nm. Several other light sources with wider spectral range were trialed in the measurement, but poorer signal-to-noise ratio compared with the stronger EDFA output made some of the spectra difficult to observe. The EDFA has a narrower spectral range but much higher signal-to-noise allowing rejection dips <1 dB strength to be readily tracked. The tracked growth curves of the spectral “dips” or notches during the fabrication and the transmission spectrum after inscription of one seed LPG are presented in Figure 1a,b, in which the rejection strength of the LPG is labeled as *R*.

**Figure 2:**
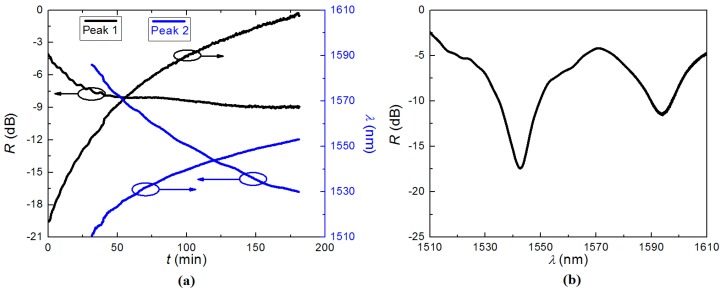
(**a**) The evolution of resonant wavelength, *λ*, and strength, *R*, of the resonant dips of the seed LPG at room temperature; (**b**) transmission spectrum of the LPG after 2 h.

Due to the out-diffusion of hydrogen with ongoing exposure, and the ultra-high sensitivity of LPG to transverse changes, six resonant dips appeared and vanished, while only one resonant dip was expected in the 100 nm spectral range for LPGs fabricated in non-hydrogen-loaded fibre with comparable period. The final transmission notch was located at *λ*_LPG_ ~ 1520 nm with a rejection strength of *R* ~ 4 dB when the UV exposure stopped. The transmission spectrum of one seed LPG was monitored at room temperature (*T* = 25 °C) for *t* = 3 h without any subsequent processing. The evolution of the resonant wavelength, *λ*_LPG_, and the rejection strength, *R*, of the resonant notches at room temperature are presented in Figure 2a, and the transmission spectrum of the LPG after 2 h, a typical result in this process is shown in Figure 2b. The evolution of the LPG during and after fabrication can be seen at [26].

During the first 30 min, only one resonant dip is observed over the measured wavelength range. The resonant wavelength, *λ*_LPG_, experienced a move towards longer wavelengths from less than 1520 nm to about 1550 nm during growth. Half an hour later, another resonant dip appears—*λ*_LPG_ moved to longer wavelengths more slowly while the strength grew much more rapidly compared with the first dip.

After 3 h, the first dip moved to *λ*_1_ ~ 1610 nm with almost stable strength of *R* ~ 9 dB, and the second dip was located at *λ*_2_ ~ 1550 nm with strength *R* ~ 15 dB. The limits of spectral range may play a part in the unusual growth of the LPGs. We have tried to use a broadband white light source to get more information, but it was difficult to track the LPG in the complex spectra of our white light source, which also was much weaker and closer to the noise floor. More likely, the transmission spectra changes are the result of out-diffusion of hydrogen, similar to those reported previously for conventionally written fibre LPGs [27]. For FBG fabrication, the hydrogen out-diffusion leads to a blue shift in FBG wavelength—part of the reason for annealing FBGs is to accelerate and remove the hydrogen. For LPG fabrication, since the LPG is determined by the coupling between core and cladding modes, the out-diffusion of hydrogen can impact evolution significantly. It changes the internal pressures in the fibre, and the mismatch of the impact to the core and inner cladding can contribute to new transmission notches with varying strengths. They coincide with the 4-step dynamic evolution of hydrogen-loaded LPGs after fabrication [28]. 

### 2.2. LPG Regeneration

A computer controlled oven was used for LPG regeneration, and a type *K* thermocouple was set inside the oven to measure the real-time temperature of the oven hot zone (*L* = 10 cm). The seed LPG was placed in the middle of this zone such that the temperature distribution could be considered as uniform. The two ends of the grating are fixed onto stages on both sides and placed under tension with a load of *m* ~ 3.5 g. From the evolution curves in Figure 2a, when the annealing for regeneration began about 30 min after fabrication, the transmission spectrum of the seed LPG changed fractionally, with a small red shift in *λ*_LPG_ and a slight growth in *R*.

**Figure 3:**
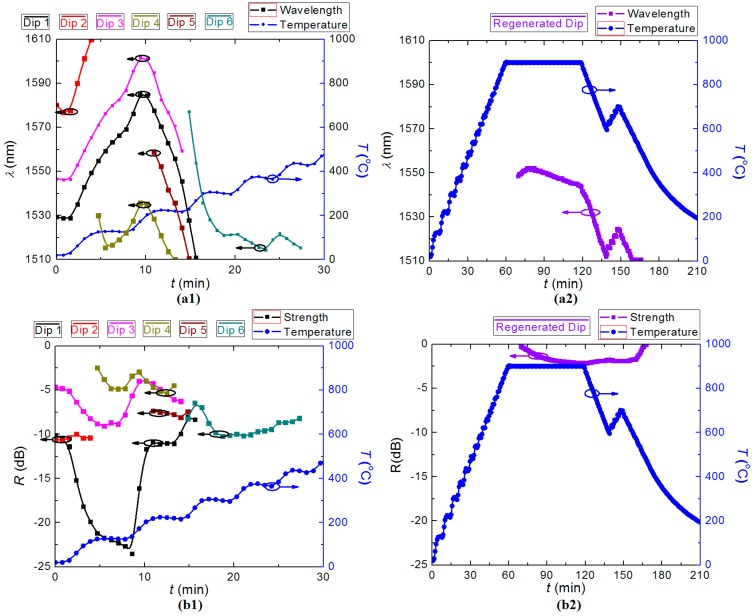
Evolution of the rejection dip during LPG regeneration: (**a1**) resonant wavelength, *λ*_LPG_; and (**b1**) rejection strength, *R*, of the LPG during the seed grating degradation; (**a2**) resonant wavelength, *λ*_LPG_; and (**b2**) rejection strength, *R*, of the LPG during regeneration.

For FBG regeneration, the elevated temperature for thermal annealing is selected between *T* = (800–950) °C, based on previous work [9]. Here, the temperature was ramped to *T* ~ 900 °C isothermally from room temperature in 1 h and then held for another h before cooling down. The transmission spectrum of a seed LPG was measured continuously during the annealing, and the evolution of the LPG is shown in Figure 3a,b.

**Figure 4:**
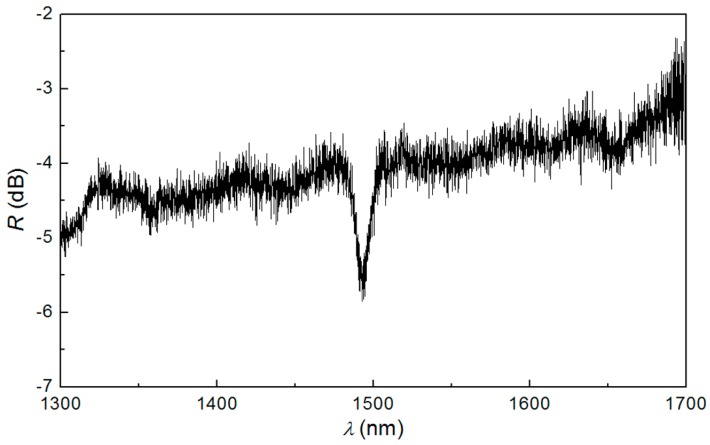
Transmission spectrum of the regenerated LPG measured with a supercontinuum source.

Initially, six transmission notches were observed with various strengths while the highest strength is *R* ~ 23 dB at *T* ~ 100 °C. From Figure 2, the variation was attributed to the accelerated hydrogen out-diffusion with increasing temperature. All the notches vanished before *T* ~ 600 °C, and the rejection strength fell below the noise floor. A regenerated notch appeared at *λ*_LPG_ ~ 1555 nm after about 70 min annealing at *T* ~ 900 °C. During this temperature dwell, the regenerated LPG slowly shifted to longer wavelengths at first with increasing strength until *R* ~ 2 dB, before moving to shorter wavelengths after the regeneration process, which arose not from regeneration but from the optical fibre itself relaxing under load after being heated up to such a temperature. This has also been observed with all fibre gratings that go up to 800 °C and stem from the stretching of the fibre due to glass softening at high temperature under the applied tension holding the fibre straight [29]. LPGs are much more sensitive to small changes than FBGs—this can be used to characterize the glass fibre properties. The regenerated LPG is not affected in terms of stable modulation or strength as shown. When the oven was cooled down, the blue shift accelerated and *R* remained stable. In order to confirm that this was a regenerated LPG, the temperature of the oven was increased from 600 °C to 700 °C, and then cooled down again. The transmission notch exhibited no deterioration and had a positive temperature coefficient of d*λ*/d*T* ~ 0.108 nm/K, showing behavior characteristic of regeneration. After the temperature fell below *T* ~ 500 °C, the regenerated LPG moved outside the wavelength span of the EDFA. The EDFA was replaced with a broadband supercontinuum source to keep track of the spectra (with lower resolution), shown in Figure 4. The ultimate regenerated LPG (RLPG) at room temperature was found at *λ*_RLPG_ ~ 1494 nm with stable rejection strength of *R* = 1.5 dB. The dip disappears when the refractive index matching gel is placed on the grating region and not elsewhere, demonstrating the existence of an LPG, which was the result of coupling between the core mode and the cladding mode.

**Figure 5:**
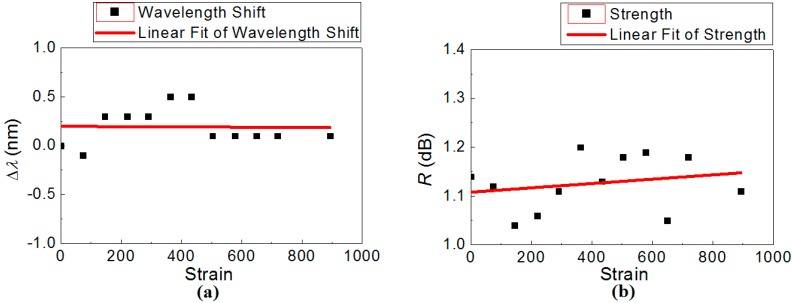
The strain dependence of the regenerated LPG: (**a**) resonant wavelength shift, Δ*λ*; and (**b**) rejection strength, *R*.

The strain sensitivity of the regenerated LPG was characterised on a translation stage by fixing one end whilst stretching the other at room temperature. Figure 5 summarises the results. A near linear dependence is obtained (Figure 5a), yielding a remarkably-low strain coefficient of *dλ/dε* ~ 1.39 × 10^−2^ pm/με up to almost 1000 με, whilst the rejection strength is quite stable with variation less than Δ*R* = ±0.1 dB, attributed to the power fluctuation of the supercontinuum light source and the resolution limitation of the OSA.

### 2.3. Regeneration through a Different Annealing Schedule

For grating regeneration, the thermal recipe plays an import role in determining the speed and efficiency of the process, particularly in doped glasses where these temperatures span compositional transition temperatures. Glass relaxation underpinning the processes can also be directly affected by thermal expansion of different layers (core, inner cladding and outer cladding) in the W fibre and the changes in interfacial stress that brings. To explore this aspect, several seed LPGs were fabricated under similar conditions but processed under different thermal recipes for regeneration.

In the first set of experiments, the thermal recipe was kept the same but the applied load on the seed LPG during regeneration was changed between *m* = 0 and *m* = 7 g. The regenerated LPGs under different loads shows nearly the same performance, including similar strain sensitivity, with the resonant wavelength variation less than Δ*λ* = ±0.1 nm. These results are similar to those in Figure 5 and establish the intrinsic insensitivity to strain for the regenerated LPGs.

**Figure 6:**
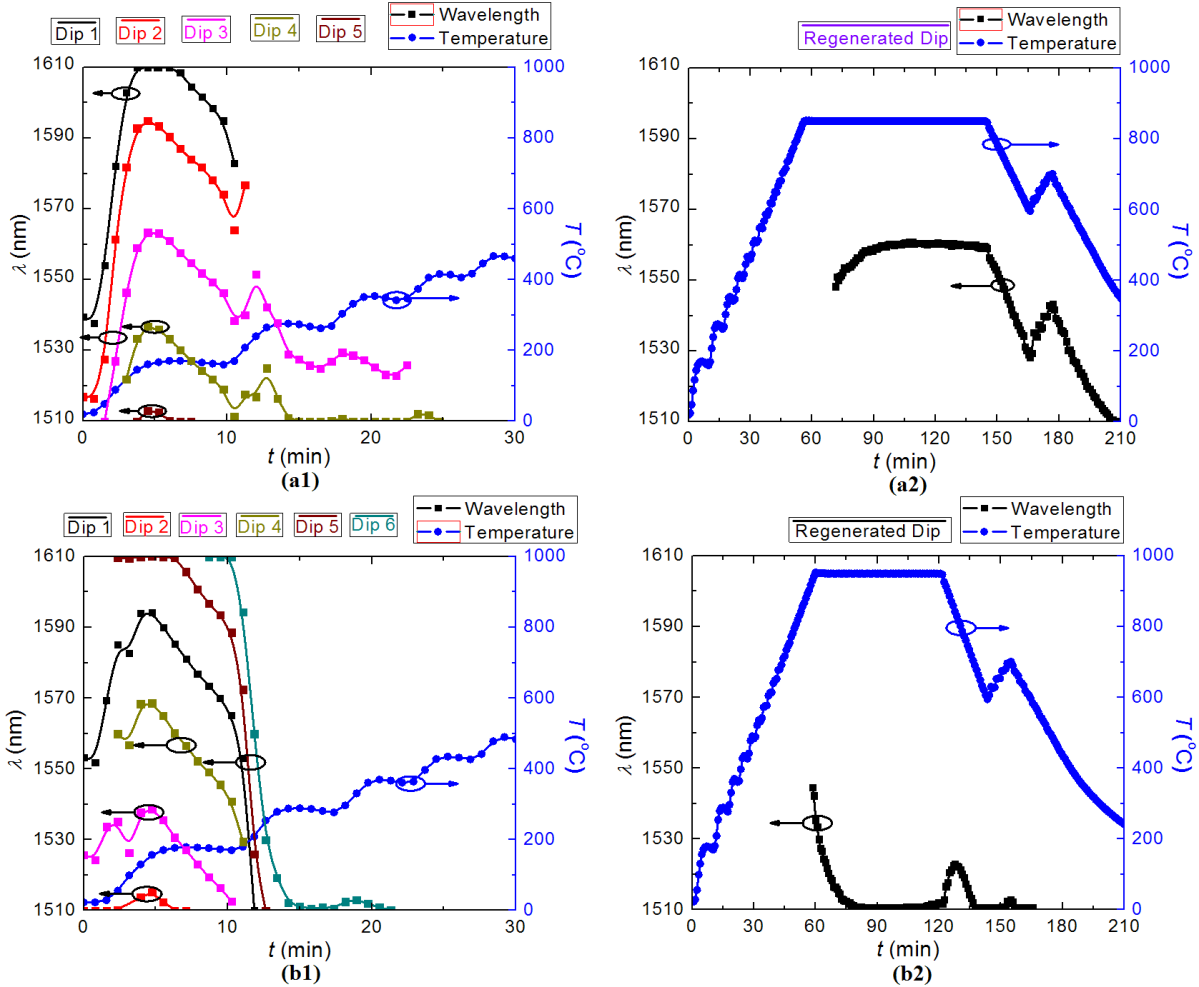
Evolution of the resonant wavelength during the LPG regeneration: (**a1**) degradation of the seed LPG, and (**a2**) regeneration at *T*_dwell_ = 850 °C; (**b1**) degradation of the seed LPG, and (**b2**) regeneration at *T*_dwell_ = 950 °C.

The regeneration dwell time was fixed to *t* = 1 h, and the same thermal recipe as shown in Figure 3 was used, with the exception that the dwell temperatures were changed to *T* = 850 °C and 950 °C between *t* = (60–120) min. The characteristic curves of *λ*_LPG_ when annealed at *T* = 850 °C and *T* = 950 °C are shown in Figure 6. The characteristics of the three regenerated LPGs have been summarised in the Table 1.

**Table 1:** Characteristics of the three regenerated LPGs dwelling at different temperatures.

*T*_reg_ (°C)	850	900	950
*t*_annealing_ before regeneration (min)	75	70	61
*t*_reg_ (min)	40	20	10
*λ*_LPG_ (nm) at *T* = 25 °C	1521	1494	1487
*R* (dB)	1	1.5	1.5
*dλ*/*dT* (nm/°C)	0.109	0.108	0.087
*dλ/dε* (pm/με)	−0.584	−0.014	−0.144

Figure 6 shows LPG regeneration at different annealing temperatures. It is observed that when temperature was fixed for a finite period, *λ*_LPG_ shifts varied: at *T* = 850 °C *λ*_LPG_ shifted to longer wavelengths at first and then became stable whereas at *T* = 950 °C *λ*_LPG_ shifted to shorter wavelengths, which showed the obvious fibre relaxation at high temperature over 800 °C and will be discussed in Section 3. When the temperature decreased below *T* ~ 860 °C in Figure 6b2, the regenerated LPG uncharacteristically shifted to longer wavelengths, compared with the other two regenerated LPGs. Figure 6 and Table 1 also show that the annealing time before regeneration occurred is shorter and it is at a shorter *λ*_LPG_ when a higher dwell temperature was set. It also demonstrates that, similar to FBG regeneration, changing the dwell temperature is an effective method to tune the resonant wavelength of the regenerated LPG. The evolution of LPG regeneration for seed gratings under different thermal recipes can be watched online at [30,31,32,33]. 

### 2.4. Post-Annealing

**Figure 7:**
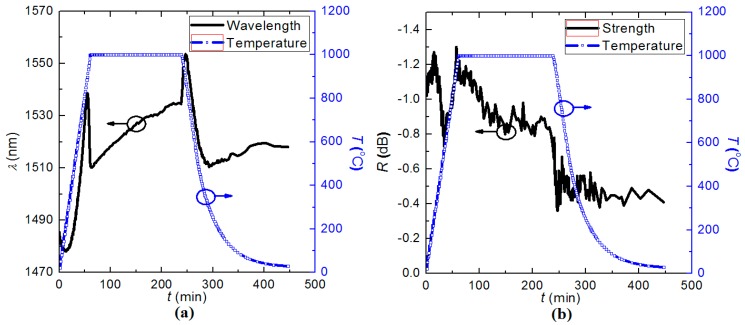
Evolution of the resonant dips during the post-annealing: (**a**) Resonant wavelength, *λ*_LPG_; and (**b**) Rejection strength, *R*.

To test the thermal stability of regenerated LPGs over 1000 °C, the three regenerated LPGs in Table 1 were post-annealed. The oven was heated to 1000 °C in 1 h, and then dwelled for 3 h at this temperature before cooling down. Transmission spectra of the regenerated LPGs were monitored continuously using the supercontinuum light source and OSA. From the results in Table 1, the LPG regenerated at higher temperature should stabilise faster. If the thermal annealing is long enough for fibre relaxation to reach equilibrium, the LPG regenerated at *T* = 950 °C should stabilise first. The evolution of the *λ*_LPG_ and *R* are shown in Figure 7.

Figure 7a suggests that the regenerated LPG has a complex, non-linear temperature dependence and the relatively sudden changes at the beginning and end of the regeneration temperature are not observed in FBG regeneration. Initially, the LPG has a slightly negative temperature coefficient below *T* ~ 250 °C, after which there is a positive dependence between 250 °C and 900 °C. Towards the end of this period *λ*_LPG_ shifted to shorter wavelength, notably with increasing temperature over the range (900–1000) °C.

**Figure 8:**
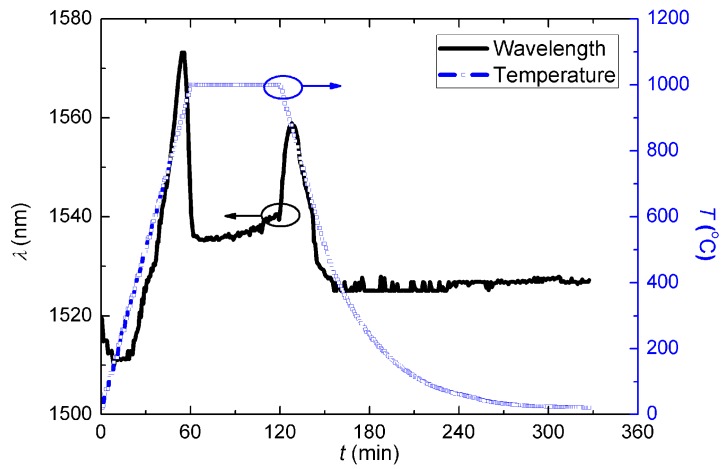
Evolution of the resonant wavelength, *λ*_LPG_, during the second post-annealing.

When dwelling at *T* = 1000 °C, *λ*_LPG_ again slowly moves to longer wavelengths. During the cooling down above *T* = 250 °C, the regenerated LPG exhibited a similar wavelength jump as that in the heating process. The resonant wavelength exhibited a jump to longer wavelengths before decreasing until slowly increasing after *T* = 500 °C. *λ*_LPG_ was ~1518 nm at room temperature, which was about 40 nm longer than prior to post-annealing. Figure 7b shows the evolution of the rejection strength. *R* grew a small amount below *T* = 200 °C before decreasing heavily between 250 and 500 °C. It then recovered as the temperature rose to *T* = 1000 °C. During the dwell period, the grating strength decreased from *R* ~ 1.2 dB to *R* ~ 0.8 dB gradually. Upon cooling back to room temperature, it decreased to *R* ~ 0.5 dB fluctuating dramatically indicating some mechanical relaxation oscillations may be occurring at the core-cladding interface. The complex behavior for both the wavelength and the rejection strengths may arise from mode hopping between core and different cladding modes but it seems another contribution is responsible for this complexity. An additional post-annealing step was applied to the LPG regenerated at 950 °C in order to explore its subsequent thermal stability. Here, the dwell time at 1000 °C was changed to *t* = 1 h, whilst the remaining steps were identical to the thermal recipe shown in Figure 7. The evolution of *λ*_LPG_ is summarised in Figure 8.

Similar to the first post-annealing process, it shows a complex, non-linear temperature dependence. *λ*_LPG_ has two similar jumps just before *t* = 60 min and again after *t* = 120 min. When the dwell temperature was at *T* = 1000 °C, *λ*_LPG_ shifted to longer wavelengths slowly. During the whole process, *R* ~ (0.5 ± 0.1) dB. Upon cooling back to room temperature, the *λ*_LPG_ has not recovered and instead is at longer wavelengths. 

**Figure 9:**
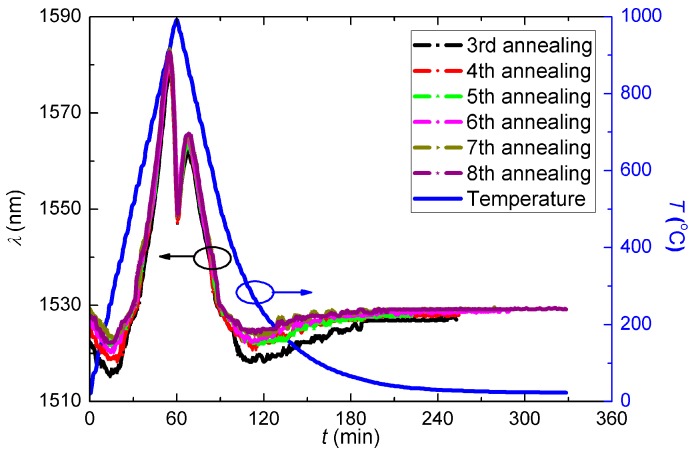
Evolution curves during the 6 times thermal annealing scanning procedures.

To explore high temperature stabilisation of the waveguide structure, six more post-annealing processes were carried out on the same grating, in this case with no dwell temperature—the temperature was scanned from room temperature to 1000 °C in 1 h and immediately cooled down so in this case there is no dwell time. The evolution curves during these thermal annealing procedures are shown in Figure 9.

**Figure 10:**
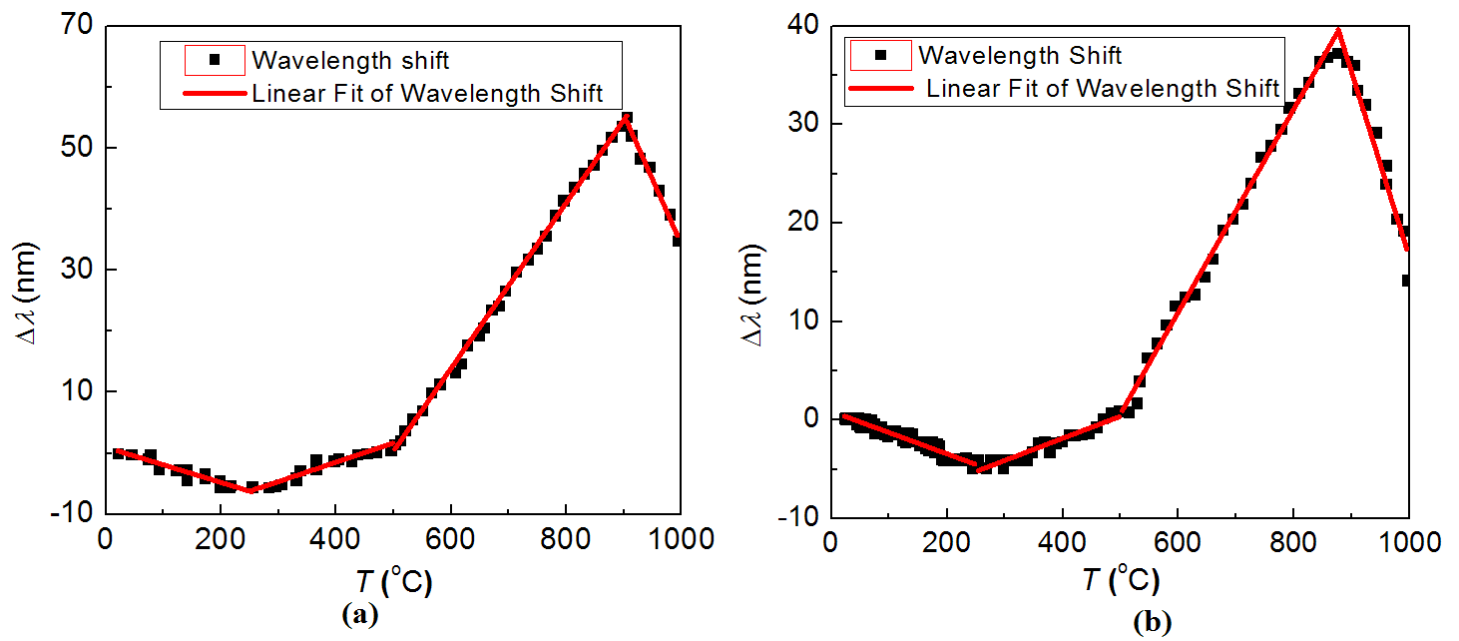
Temperature dependence of the regenerated LPG after stabilisation: with (**a**) increasing temperature; and (**b**) decreasing temperature.

It illustrates that, after about four of the further annealing steps, the regenerated LPG was eventually stabilised. At the fifth annealing step, the *λ*_LPG_ before and after annealing were almost the same, and the last two evolution curves nearly overlapped with each other exactly. The stabilised resonant wavelength was *λ*_LPG_ ~ 1529 nm at room temperature with strength of *R* ~ 0.5 dB. It is apparent that, the temperature dependencies of the heating and cooling steps are different, as the maximum value of *λ*_LPG_ in the two steps are ~1582 nm @ 900 °C and ~1565 nm @ 870 °C, respectively. When analysed closely, the annealing curve can be examined by dividing the evolution into piece-wise linear fitting, as shown in Figure 10, where the fitting functions are presented as:

$$\Delta \lambda_{1}=\left\{ \begin{array}{l} -0.02881T+0.9883\;\;\;\;\;\;\;\;(20{}^{\circ}C\leq T\leq 250{}^{\circ}C)\\ 0.02891T-13.40\;\;\;\;\;\;\;\;\;\;\;\;(250{}^{\circ}C\leq T\leq 500{}^{\circ}C)\\ 0.1356T-67.37\;\;\;\;\;\;\;\;\;\;\;\;\;(500{}^{\circ}C\leq T\leq 900{}^{\circ}C)\\ -0.2121T+246.3\;\;\;\;\;\;\;\;\;\;\;(900{}^{\circ}C\leq T\leq 1000{}^{\circ}C) \end{array}\right.\;\;(\text{Increasing temperature})$$

$$\Delta \lambda_{2}=\left\{ \begin{array}{l} -0.02155T+0.8808\;\;\;\;\;\;\;(20{}^{\circ}C\leq T\leq 250{}^{\circ}C)\\ 0.02248T-10.85\;\;\;\;\;\;\;\;\;\;\;(250{}^{\circ}C\leq T\leq 500{}^{\circ}C)\\ 0.1033T-51.02\;\;\;\;\;\;\;\;\;\;\;\;\;(500{}^{\circ}C\leq T\leq 870{}^{\circ}C)\\ -0.1878T+204.2\;\;\;\;\;\;\;\;\;\;(870{}^{\circ}C\leq T\leq 1000{}^{\circ}C) \end{array}\right.\;\;(\text{Decreasing temperature})$$

Through similar post-annealing schedules, the other two regenerated LPGs were stabilised, and the temperature coefficients are characterised with similar piece-wise linear fitting. The results are presented below. The details for the post-annealing of the three LPGs are listed in Table 2.

**Table 2:** Post-annealing details and characteristics of the three stabilised regenerated LPGs.

Regeneration Temperature (°C)	850	900	950
*t*_dwell_ 1000 °C (h)	6	5	4
No. of annealing steps	5	4	4
*λ*_LPG_ (nm) @ *T* = 25 °C	1545	1533	1529
*R* (dB)	0.4	0.5	0.5
*dλ/dε* (pm/με)	−0.397	−0.082	−0.105

For LPG Regenerated at *T* = 900 °C:

$$\Delta \lambda_{1}=\left\{ \begin{array}{l} -0.01790T-0.2335\;\;\;\;\;\;\;\;(20{}^{\circ}C\leq T\leq 250{}^{\circ}C)\\ 0.02461T-10.97\;\;\;\;\;\;\;\;\;\;\;\;(250{}^{\circ}C\leq T\leq 500{}^{\circ}C)\\ 0.09799T-43.84\;\;\;\;\;\;\;\;\;\;\;\;\;(500{}^{\circ}C\leq T\leq 890{}^{\circ}C)\\ -0.1975T+211.7\;\;\;\;\;\;\;\;\;\;\;(890{}^{\circ}C\leq T\leq 1000{}^{\circ}C) \end{array}\right.\;\;(\text{Increasing temperature})$$

$$\Delta \lambda_{2}=\left\{ \begin{array}{l} -0.01798T-0.7644\;\;\;\;\;\;\;(20{}^{\circ}C\leq T\leq 250{}^{\circ}C)\\ 0.02235T-10.76\;\;\;\;\;\;\;\;\;\;\;(250{}^{\circ}C\leq T\leq 500{}^{\circ}C)\\ 0.09877T-44.96\;\;\;\;\;\;\;\;\;\;\;\;\;(500{}^{\circ}C\leq T\leq 860{}^{\circ}C)\\ -0.1898T+204.2\;\;\;\;\;\;\;\;\;\;(860{}^{\circ}C\leq T\leq 1000{}^{\circ}C) \end{array}\right.\;\;(\text{Decreasing temperature})$$

For LPG Regenerated at *T* = 850 °C:

$$\Delta \lambda_{1}=\left\{ \begin{array}{l} -0.02802T-0.1294\;\;\;\;\;\;\;\;(20{}^{\circ}C\leq T\leq 250{}^{\circ}C)\\ 0.04409T-18.75\;\;\;\;\;\;\;\;\;\;\;\;(250{}^{\circ}C\leq T\leq 500{}^{\circ}C)\\ 0.1282T-62.37\;\;\;\;\;\;\;\;\;\;\;\;\;(500{}^{\circ}C\leq T\leq 900{}^{\circ}C)\\ -0.3380T+359.3\;\;\;\;\;\;\;\;\;\;\;(900{}^{\circ}C\leq T\leq 1000{}^{\circ}C) \end{array}\right.\;\;(\text{Increasing temperature})$$

$$\Delta \lambda_{2}=\left\{ \begin{array}{l} -0.02674T+0.9070\;\;\;\;\;\;\;(20{}^{\circ}C\leq T\leq 250{}^{\circ}C)\\ 0.03139T-13.78\;\;\;\;\;\;\;\;\;\;\;(250{}^{\circ}C\leq T\leq 500{}^{\circ}C)\\ 0.09973T-46.64\;\;\;\;\;\;\;\;\;\;\;\;\;(500{}^{\circ}C\leq T\leq 870{}^{\circ}C)\\ -0.1959T+212.9\;\;\;\;\;\;\;\;\;\;(870{}^{\circ}C\leq T\leq 1000{}^{\circ}C) \end{array}\right.\;\;(\text{Decreasing temperature})$$

The results are for the most part as expected, for example indicating that it requires a much longer post-annealing stage to stabilise the LPG regenerated at lower temperatures than at higher temperatures. The LPGs have a longer wavelength as well. LPG strain sensitivity was not found to differ after post-annealing—the small variations may be explained as arising from changes in waveguiding due to glass relaxation during post-annealing, in addition to light source fluctuations and the OSA resolution. Striking differences are observed at 900 and 950 °C, where the effective strain optic coefficients appear to have reduced significantly (near zero at 900 °C) suggesting significant changes arising from the balance between grating pitch and mode overlap, stress annealing and ultimately glass relaxation. Over 10 LPGs were successfully regenerated under different conditions, demonstrating the feasibility, control and reproducibility as well as reliability of the technique.

Although the regenerated LPGs reported here are not strong, the basis for much stronger regenerated LPGs through optimization of both fibre design and materials has been established. It is worth pointing out that the first regenerated FBGs were quite weak [6], but now regenerated FBGs are strong enough for regenerated fibre lasers [34], and applied in real field tests, such as diesel locomotive turbine temperature diagnostics [23]. What’s more, for distributed high temperature applications such as those involving aircraft and vehicular interrogation where low resolution and low cost lightweight systems are needed arrays of weak LPGs are required to tap off a small amount of light and to equalize system performances. The same applies to any spectroscopic interrogation within an industrial setting which required either low resolution filters or with spectral to intensity conversion using the LPG edge as a ramp with which to probe FBG shifts due to strain or temperature. In contrast to weak narrowband FBGs used extensively in field applications, LPGs greatly relax interrogator and source requirements and costs because they are broad and not narrow, making them amenable to chirped ramp filter designs for these same interrogators. To have them performing in high temperature environments where only a few or an array of regenerated LPGs are required is ideal. 

## 3. Discussion

The observed differences between regenerated LPGs and previously reported regenerated FBGs rest entirely with the mode coupling differences of the two structures where the LPG involves coupling between transversely distinct forward traveling modes and the FBG coupling between transversely invariant forward and backward traveling modes. The regenerated LPGs show reduced strain sensitivity compared to the pristine seed LPG. Applying different but uniform loads during regeneration within the W fibre does not significantly affect the regenerated LPG spectra, a response distinct to that observed with regenerated FBGs [29] which suggests the changes seen by both cladding and core modes are similar. It is explained by the much greater contribution to the changes in the inner cladding of the W fibre for LPGs; these changes are seen much more strongly by the cladding mode. The strain sensitivity of the LPG is determined by both waveguide and material contributions and adjusting these parameters by appropriate fibre and/or grating pitch design, for example, can make it positive, negative or zero. The near zero strain sensitivity makes the regenerated LPGs in W fibres ideal candidates for high temperature refractive index sensing since they are immune to the influence of applied load.

Both regenerated FBGs and LPGs need post-annealing to stabilise for ultra-high temperature measurements. In contrast to the rapid FBG regeneration and stabilisation [6], it takes more than 5 h for regenerated LPGs to be similarly stabilised, in part because the core-cladding coupling mechanism is much more sensitive to smaller changes in the fibre than the FBG backwards and forward coupled traveling modes. As mentioned above, the single mode fibre used for grating fabrication has a complex W structure and can be considered a composite material system. It has a lower refractive index inner cladding through doping with P and F, along with the dopants of Ge and B in the core. Although the structural transformation of silica, rather than the dopants, is key to the high temperature stability of the regenerated structures during and after thermal annealing, the dopants do affect the thermal coefficients of the core and inner cladding, which correlate with differences in glass relaxation between the two layers as well as differences in the stress fields that contribute to glass memory differentiation upon which regeneration depends. Table 3 provides the thermal expansion coefficients (*α*), transition temperatures (*T*g) and melting temperatures (*T*m) of different components in the fibre [35,36,37,38,39,40]. The thermal coefficients of fluorine are not listed as it does not exist as an oxide in the fibre, but fluorine doping leads to a drastic drop in glass transition by displacing oxygen [41,42].

**Table 3:** Thermal coefficient of different components in the fibre.

	SiO_2_	B_2_O_3_	GeO_2_	P_2_O_5_	F
*α* (×10^−7^/°C)	4.1	151	64	13.8	/
*T*g (°C)	~1173	~270	~530	~264	/
*T*m (°C)	1600	450	1115	340	/
Existence	All the three layers	Core		Inner cladding	

From Table 3, the dopants generally decrease the transition temperature and melting temperature, while increasing the expansion coefficients in the core and inner cladding. It can be deduced that, on account of the much lower transition temperature of dopants and the rather noticeable proportion in the fibre composition, the transition and softening temperatures of the core and inner cladding should be much lower than 850 °C. This leads to the observation of nanoscale dimensional change in the core and inner cladding of conventional LPGs fabricated in the same kind of fibre through relative low temperature annealing (*T* = 300 °C, *t* = 1 h) [43]. In fact in that work it is precisely the additional sensitivity to structural self-monitoring that allows such changes to be inferred. The core region has the largest thermal expansion coefficient, whilst the outer cladding has the lowest. Consequently, when the preform is drawn to fibre, the inner layer has larger shrinkage than the outer layer during the rapid quenching, leading to large tensile stresses.

Whereas the LPGs regenerated at different temperatures show similar performances during the post-annealing, the different spectral shift of the resonant wavelength, *λ*_LPG_, is not readily explained by coupling of different cladding modes, in spite of the dependence of the temperature coefficient on mode selection. More likely, the difference arises from the thermal characteristics of the fibre. When the regenerated LPG is processed through post-annealing, other unusual observations are made. The transmission notch shifts smoothly and the strain sensitivities of all the three regenerated LPGs do not show a large difference through annealing—this is consistent with a specific cladding mode coupling with the core mode. However, the temperature response is not linear and had to be fitted with a piece-wise linear curve. There are clear turning-points, or threshold-like, temperatures. According to the evolution of the three LPGs regenerated at different temperatures, the turning point temperatures are: *T*_1_ ~ 250 °C, *T*_2_ ~ 500 °C, *T*_3_ ∈ (860, 900) °C. Because the coupling modes do not change during the thermal annealing, these turning-point temperatures should only be due to the thermal characteristics of the glass. It is interesting that for the three turning-point temperatures, *T*_1_ and *T*_2_ appear as constant and relatively low, but *T*_3_ is variable between 860 °C and 900 °C. This suggests that *T*_1_ and *T*_2_ are related while *T*_3_ describes something else. The transition temperature of the core or inner cladding is much lower than the melting temperature and in fact *T*_1_ and *T*_2_ coincide with the transitions of phosphate and germanate respectively. In terms of a mixed state of the glass, the higher value of *T*_3_ is consistent with the inner cladding composite where the glass softening of silica are lowered substantially; *T*_3_ coincides with the effective melting point of the fluorine co-doped phosphosilicate inner cladding: *T*_m(clad)_ = (860–900) °C. Therefore, the transition temperatures of the core and inner cladding may be identified from these experiments as around *T*_g(core)_ = 500 °C and *T*_g(clad)_ = 250 °C, respectively.

For LPG regeneration, the coupling of the core mode and the cladding mode makes LPG ultra-sensitive to changes in waveguiding properties particularly in the transverse dimension, whilst the softening and structural changes of the inner cladding at the high dwell temperature induce dimensional change and extra stress formation. When the dwell temperature is 850 °C, the inner cladding of the LPG remains solid, as the core has a larger thermal expansion coefficient, the core expands at the interface between the two layers. By contrast, when the dwell temperature is over 900 °C, the inner cladding is softened if not melted (depending on the induced pressure constraints), and the volume of this layer increases. Since the outer cladding remains stiff and inelastic, the inner cladding expands inwards compressing the softer core (and reducing the tensile stresses). As a result, when the temperature dwells above and below 900 °C during regeneration, distinct changes in spectral shift arise.

During post-annealing, the dwell temperature of 1000 °C is high enough for interfacial stress relief, but when the temperature cools down, the solidification of the inner cladding makes its volume decrease, and induces extra stress which breaks the equilibrium built up at the former annealing process. The induced stress can be annealed-out by additional annealing, but it requires a much longer time compared with regenerated FBG stabilisation. 

When the temperature is below the melting point of the inner cladding, both the core and inner cladding are highly viscous. Similar to the dwell process during regeneration, the densification of the inner cladding increases the refractive index whilst the thermal coefficient is smaller than that in the core region due to doping. As a result, the temperature dependence of the regenerated LPG changes direction. When the temperature drops to below 500 °C, the core region exhibits a phase transition, so the temperature dependence changes and another turning point occurs. As the volume of the core is rather small relative to the inner cladding, the temperature dependence decreases due to changes in the core, but does not change direction. However, when the temperature is below 250 °C, the large volume of the inner cladding and the rising stress is significant enough to change the temperature coefficient direction again. The temperature dependencies of the heating and cooling steps are different, as the impacts of the glass relaxation in the heating and cooling steps are not the same, and correlate to the equilibrium built up among the two layers throughout their thermal history, which is particularly obvious at the melting temperature of the inner cladding with different pressure constraints between melting and solidification process.

In summary, the difference in coupling between short period FBGs and long period LPGs gives rise to differences in regenerated device properties generally. The LPGs experience significant changes in glass properties between core and inner cladding that can affect the direction of spectral shift because the coupling occurs between core and cladding modes. Paradoxically, within W design fibres this can offset mechanical imposts on the grating making them strain insensitive. These remarkable properties of complex trimaterial glass systems combined with extremely sensitive spatial interrogation possible through waveguide modes both in the core and cladding interacting over very large distances at the interface of the materials, offers unprecedented opportunities for fundamental glass studies. Through LPG regeneration and post-annealing, the performance of the regenerated LPG shows complex, as exploitable, details that are not seen with regenerated FBGs—the two systems can be used in conjunction to solidify the spatially dependent differences between the two systems. All this information can be used to directly optimise novel glass configurations and understand exactly what they do at least in optical fibre form and lay the ground work for predictive modelling of more complex glassy structures.

## 4. Conclusions

The feasibility of mass production and reproducibility of LPG regeneration is demonstrated and the performance of the LPGs during regeneration and subsequent post-annealing were investigated in depth. For LPGs regenerated at different temperatures, all of them exhibit reduced strain sensitivity and piece-wise temperature dependence. Complex spectral behavior is found to be dependent on the temperatures used with red shifts and blue shifts observed below and above a core softening or melting temperature. These behaviors are consistent with the differences in glass properties arising from the difference in glass composition of the core and inner cladding of the W fibre design. Based on the measurement of the stabilised regenerated LPGs, the transition temperatures of the core and the inner cladding are estimated near *T*_g(core)_ = 500 °C and *T*_g(clad)_ *=* 250 °C and the melting temperature of the inner cladding is *T*_m(clad)_ ~ (860–900) °C. These methods show how photonic in-waveguide components can be used as new tools for in-depth composite glass studies revealing new insights with unprecedented sensitivity. 
